# Challenges in the Management of Bradycardia, Renal Failure, Atrioventricular Blockade, Shock, and Hyperkalemia (BRASH) Syndrome in an Elderly Patient: A Case Report

**DOI:** 10.7759/cureus.82364

**Published:** 2025-04-16

**Authors:** Marwa Morgom, Moayad A Elgassim, Sujood F Awadelseed, Leena Saeed

**Affiliations:** 1 Emergency Medicine, Hamad Medical Corporation, Doha, QAT; 2 Medical Education, Hamad Medical Corporation, Doha, QAT; 3 Medical Research Center, Hamad Medical Corporation, Doha, QAT

**Keywords:** bradycardia, brash syndrome, geriatric, heart failure, hypotension, kidney injury

## Abstract

This case report describes the clinical course of a 77-year-old woman with a complex medical history, including atrial fibrillation, hypertension, coronary artery disease, heart failure, and cognitive impairment. Despite multiple hospitalizations and interventions, her condition deteriorated, ultimately leading to death. The patient's most recent hospitalization was for the management of bradycardia, renal failure, atrioventricular node blocker, shock, and hyperkalemia (BRASH) syndrome. Aggressive medical interventions, including pressor support and non-invasive ventilation, were unable to stabilize her condition. This case highlights the challenges associated with managing elderly patients with multiple comorbidities, particularly when faced with severe bradycardia and hypotension.

## Introduction

BRASH syndrome, a newly recognized clinical condition, is defined by a combination of five key symptoms: bradycardia, renal failure, atrioventricular (AV) block, shock, and hyperkalemia [1]. This condition is typically seen in elderly patients with heart disease and compromised kidney function, as it is associated with the use of antihypertensive medications and diminished renal capacity [2]. The recognition of bradycardia in BRASH syndrome has significant clinical implications, as it can present more severely than expected and may be resistant to standard treatment approaches. This can lead to hemodynamic instability and worsen patient outcomes [3].

This case report presents the clinical course of a 77-year-old female with a complex medical history, including atrial fibrillation, hypertension, coronary artery disease, heart failure, and cognitive impairment. Her advanced age and multiple comorbidities posed significant challenges for her care. Despite extensive medical interventions and hospitalizations, her condition deteriorated over time, ultimately leading to death.

This case report provides insights into the challenges associated with managing elderly patients with multiple comorbidities, especially in the context of limited communication and complex medical decisions. It also highlights the importance of comprehensive care planning, including consideration of end-of-life care, for patients with advanced medical conditions.

## Case presentation

A 77-year-old female, with a known case of atrial fibrillation, hypertension, coronary artery disease in the 1980s not stented, heart failure with mild reduced ejection fraction, diastolic dysfunction, and left ventricular hypertrophy (LVF). Her last ejection fraction (EF) was 47%. She is on apixaban and metoprolol 100 mg BID. She has visual, auditory, and memory impairment, recurrent GI bleeding, last in 3/2023, treated conservatively. The pros and cons of anticoagulation were discussed with the patient's family, and they decided to continue anticoagulation.

The patient was recently admitted for around two weeks under the medical team with the diagnosis of acute-on-chronic type 2 respiratory failure with respiratory acidosis due to infection and volume overload. Her baseline functional status is fully bedbound, noncommunicating (occasionally open eyes in response to commands), on nasogastric (NG) feed, using BiPAP/oxygen (usually 1 L nasal cannula) at home. Apixaban was discontinued during this admission due to an elevated risk of bleeding, following the patient's recent presentation with hematuria. After a thorough discussion with the patient's family regarding the potential risks and benefits of continuing the medication, a decision was made to stop it. The patient was subsequently discharged with a do-not-attempt-resuscitation (DNAR) code status.

The day after discharge, the patient was brought again by family as they noticed an episode of sweating after feeding (she is taking 100 ml five times a day by syringe through mouth) and dyspnea with desaturation to around 78-80%. No fever, cough, or increase in secretion was noted. Upon arrival of the emergency medical services (EMS), the patient was found to be desaturating, connected to 15 L oxygen via a non-rebreather mask, then continuous positive airway pressure (CPAP). She was also found to be hypertensive, with a systolic blood pressure (SBP) of 168. Glyceryl trinitrate (GTN) spray was given, and an intraosseous line (IO) was inserted. Upon arrival at the emergency department (ED), the patient was tachypneic and tachycardic up to 140 bpm, hypotensive, with a BP of 88/60, and a mean arterial pressure (MAP) of 69. A bolus of normal saline (500 ml) was given via the IO line. The patient was also connected to noninvasive ventilation (NIV) for hypoxia and hypercapnia and given one dose of ceftriaxone 2g. No labs were obtained as she had difficult venous access. Only arterial blood gas (ABG) was done, which suggests chronic respiratory acidosis with compensatory metabolic alkalosis (Table 1).

**Table 1:** Arterial blood gases (ABG)

Test	Result	Normal value
Blood pH	7.35	7.35 - 7.45
PCO2	79	35 - 45 mmHg
PO2	29	80 - 100 mmHg
HCO3	44.5	22 - 26 mEq/L

On examination, her pupils showed bilateral cataracts, a chest examination revealed decreased air entry on the left side without wheezes, the abdomen was distended but soft (most likely due to bilevel positive airway pressure (BiPAP)), and bilateral pitting edema extending till the abdomen with mild upper limb edema (anasarca). The impression at that time was CO2 narcosis. The patient initially required high pressures on BIPAP 32/8 with FiO2 of 50% and was admitted under the internal medicine team. They referred hypotension to the drug; home medications were resumed. No antibiotics were given at that time as no fever or other features suggestive of a new infection. Moreover, the patient recently completed a course of antibiotics in the last admission. Chest X-ray shows decreased lung volumes, a blunted left costophrenic angle (CP) angle, and no infiltrates. When compared to the last X-ray done in the last admission, the new chest X-ray showed improvement. Anesthesia was consulted for intravenous (IV) cannula insertion as it is very difficult. BIPAP was weaned off gradually to home settings in preparation to be discharged. On the third day of this admission, the patient was desaturating to 50%, tachycardic at 130 BPM, and tachypneic RR 40, diaphoretic and in distress. The patient improved immediately after the removal of the IO line and BIPAP adjustment. She was started again on home BIPAP, which she tolerated well, and was discharged after observation until noon. The family was compliant with the NIV therapy plan from the hospital and knowledgeable on BiPAP machine use and maintenance. A private routine home health team visit was arranged by the family in a regular manner.

One month later, a home care nurse discovered potassium of 6 mmol/L during routine home health monitoring, and the family was contacted to take the patient to the ED. The family reported no fever, shortness of breath, cough, vomiting, diarrhea, urinary tract infection symptoms, or any new complaints. The patient is compliant with her NIV therapy at home. Her baseline pCO2 was around 75 mmHg. Upon evaluation in the ED, she was found to have acidosis in the venous blood gas (VBG), and repeated potassium was 6.2 mmol/L. Patient received insulin 10 units in 50% dextrose infusion, and salbutamol 5 mg nebulizer repeated twice, and IV Lasix 40 mg IV shot, her potassium improved to 4.3, but on repeated VBG, potassium was 5 mmol/L. The patient was readmitted under the medical team and resumed her home medications except spironolactone. On the second day, the patient appeared drowsy, uncomfortable, and generalized edema was noted. The swelling of the right leg was slightly more tense than the left leg. The skin was cold peripherally, and the toes were slightly darker than the usual skin color bilaterally. The vascular team was consulted, and they excluded acute artery disease, and they recommended anticoagulation if no contraindication. Heartsounds could not be heard clearly due to NIV and body habitus; the pulse felt irregular. ECG showed new changes with T-wave inversion at V1, V2, V3, and V4 leads, with possible right bundle branch block for cardiology evaluation. A new chest X-ray showed no new infiltration, NG tube in situ with longer-standing all left basal haziness. Doppler US shows no evidence of deep vein thrombosis (DVT) in the right lower limb. The nephrology consultation emphasized the need for hematuria workup as an outpatient.

Twenty days after discharge, the patient was brought to the ED after the home nurse recorded a critical lab result of low Na. In the ED, the patient had the same baseline Glasgow Coma Scale (GCS) of 4, Na was 107, and BP was 170/100. Sodium chloride 3% 100ml was given, then repeated Na became 109. Other investigations showed low chloride, high creatinine, high C-reactive protein (CRP), high alkaline phosphatase, critically high urea, and high WBC (Table 2). Chest X-ray showed no changes compared to the last X-ray.

**Table 2:** Blood test results

Test	Result	Normal value
Sodium	107	135-145 mEq/L
Chloride	69	96-106 mmol/L
Creatinine	148	62-106 umol/L
C-reactive protein	42.9	0-5
Alkaline phosphatase	194	40-129 U/L
Urea	37.8	2.5-7.8 mmol/L
White blood ccells	18.8	4-10 x 103/uL

Patient was admitted to the medical intensive care unit (MICU) for two days to manage hyponatremic/hypervolemia, then stepped down to the medicine ward, with a central line, as the patient is having difficult peripheral cannulation, hypertonic saline continued along with furosemide. Her Na improved to 120 after 24h of correction; hypertonic saline was stopped. Initially, she had elevated inflammatory markers, no complaints, and no vital instability. Starting on Tazocin for the last three days, cultures show a negative result. As a result, the antibiotics were switched to IV Augmentin. The family refused a Foley catheter for fear of infection and refused heparin DVT prophylaxis due to hematuria. The patient was discharged home as the family requested.

Ten days post last admission, the patient was brought to the ED on BIPAP, after the family observed a bradycardia episode, and also they noticed hypotension started a few days back. In the ED, HR was 40 bpm, ECG showed bradycardia, dopamine 1 mcg/kg/min and adrenaline 0.1 mcg/kg/min were given. Dopamine was then tapered and switched off; her BP and HR were 63 to 65 on adrenaline infusion, then slowly tapered down. Vital signs monitoring in the ED is shown in Table 3.

**Table 3:** Vital signs of patient at emergency department HR - heart rate; BPM - beats per minute; BP - blood pressure; RR - respiratory rate; GCS - Glasgow Coma Scale

Date	Time	HR (bpm)	BP (mmHg)	RR (breaths/min)	SpO2 (%)	Temperature (°C)	GCS
13/07/2024	09:00	40	88/60	20	78	36.5	12
13/07/2024	10:00	63	100/70	22	85	36.8	12
13/07/2024	11:00	30	80/50	25	82	36.9	10
13/07/2024	12:00	0	0	0	0	37	4

**Figure 1:**
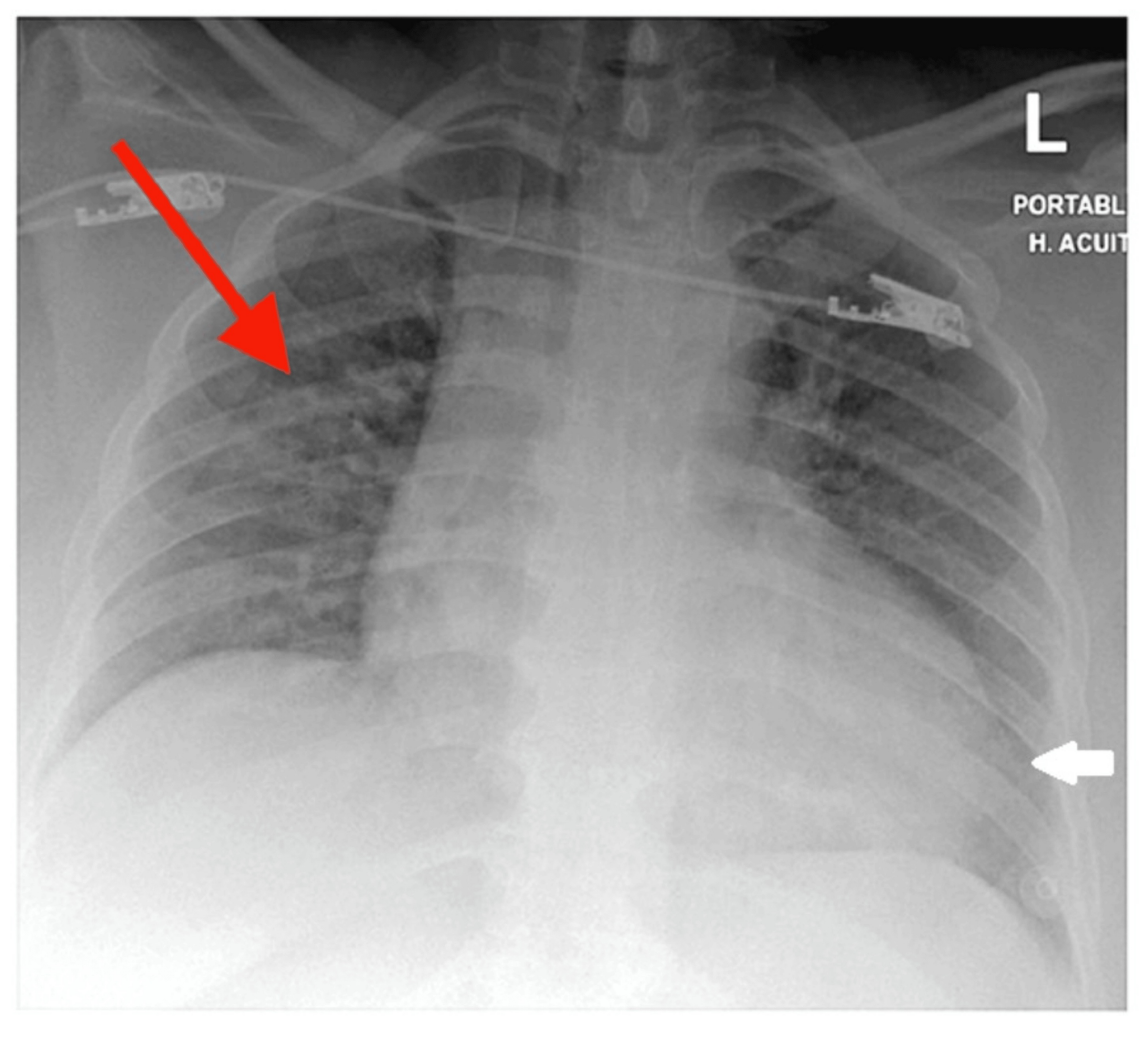
Prominent broncho-vascular markings (red arrow) and streaky shadows /haziness in the left lower zone (white arrow)

Chest X-ray revealed prominent broncho-vascular markings in the right lung and streaky shadows /haziness in the left lower zone (Figure 1). Blood tests done in the ED are reflected in Table 4.

**Table 4:** Main investigations done at the ED K - Potassium; Na - Sodium; Cl - Chloride; CRP - C-reactive protein; WBC - white blood cells; ED - emergency department

Date	Time	K (mmol/L)	Na (mmol/L)	Cl (mmol/L)	Creatinine (umol/L)	Urea (mmol/L)	CRP (mg/L)	Alkaline phosphatase (U/L)	WBC (x10^3/uL)
13/07/2024	09:00	6.2	107	69	148	37.8	42.9	194	18.8
13/07/2024	10:00	5	110	72	152	38.5	43.2	196	19
13/07/2024	11:00	4.8	112	75	155	39	43.5	198	19.2
13/07/2024	12:00	4.5	114	78	158	39.5	43.8	200	19.4

## Discussion

BRASH syndrome, an acronym for bradycardia, renal failure, atrioventricular blockade, shock, and hyperkalemia, describes a potentially life-threatening condition characterized by severe bradycardia and hyperkalemia in patients with renal impairment who are taking AV nodal blockers. This syndrome highlights the synergistic interaction between these factors, leading to significant cardiovascular complications [4].

In the presented case, the patient's history of renal insufficiency and concurrent use of an AV nodal blocker, likely a beta-blocker, created a predisposing environment for BRASH syndrome [5]. BRASH syndrome is a complex interaction between several factors that can lead to severe bradycardia and hyperkalemia. First, impaired renal function can lead to the accumulation of potassium in the blood, leading to hyperkalemia. This is particularly important in patients taking AV nodal blockers, as these medications can also affect potassium excretion. Second, AV nodal blockers such as beta-blockers and non-dihydropyridine calcium channel blockers can slow the heart rate by blocking the electrical signals that control the heart's rhythm. This can lead to bradycardia, especially in patients with underlying heart conditions or electrolyte imbalances. Third, the combination of renal insufficiency and AV nodal blocker use can create a synergistic effect, leading to more severe bradycardia and hyperkalemia than either factor alone [6].

The clinical presentation of BRASH syndrome can vary depending on the severity of bradycardia and hyperkalemia. However, common symptoms or signs often include bradycardia, which can cause dizziness, lightheadedness, fainting, fatigue, and weakness. Hyperkalemia which can present as muscle weakness, fatigue, nausea, vomiting, and in severe cases, sinus arrest and cardiac arrest [7].

In addition to these symptoms, patients with BRASH syndrome may also experience electrolyte disturbances such as hyponatremia, hypocalcemia, and hypomagnesemia. In addition, if the bradycardia is severe, it can lead to respiratory distress due to decreased oxygen delivery to the tissues. In severe cases, hyperkalemia can lead to abnormal heart rhythms, such as ventricular fibrillation, which can be fatal.

In cases of isolated hyperkalemia, hyperkalemia alone can result in bradycardia and subsequent renal failure. However, bradycardia would only result from severe hyperkalemia. This would differentiate isolated hyperkalemia from BRASH syndrome; several case reports suggest that even mild to moderate hyperkalemia can lead to severe bradycardia in patients taking AV node blockers [8]. An electrocardiogram displaying bradycardia in the absence of hyperkalemia features is an indicator of BRASH syndrome. Brash syndrome patients are also on AV nodal blockade, which isn't the case for isolated hyperkalemia [9]. Clinical history is an important factor when comparing beta-blocker toxicity to BRASH syndrome. In BRASH syndrome, patients are typically compliant with their medications and usually don't have elevated drug levels [10].

Treatment of BRASH syndrome involves correcting hyperkalemia and bradycardia. Although it may appear mild in BRASH syndrome, hyperkalemia should be promptly treated. Features of hyperkalemia on the ECG or evidence of hemodynamic instability should be treated with IV calcium. IV calcium is the front-line therapy for bradycardia as it counters hyperkalemia. High doses of potassium-wasting diuretics may be used in severe hyperkalemia to overcome diuretic resistance due to renal dysfunction. Isovolumic kaliuretic can be used to replace fluid loss due to diuretics. If patients fail to produce urine after high-dose diuretics, emergent dialysis is the definitive treatment for hyperkalemia [11].

Our patient's history of atrial fibrillation, hypertension, and coronary artery disease contributed to the development of heart failure. Her cognitive impairment, including visual, auditory, and memory deficits, further complicated her care and communication with healthcare providers. Recurrent gastrointestinal bleeding, despite conservative management, added another layer of complexity to her medical history.

The patient's admission for acute on top of chronic type 2 respiratory failure highlighted the challenges of managing her underlying medical conditions. Her baseline functional status, which included being fully bedbound, non-communicating, and dependent on NG feeding and BiPAP/oxygen therapy, further emphasized the complexity of her care. The decision to discontinue anticoagulation due to bleeding risk during her hospitalization underscored the delicate balance between managing her medical conditions and mitigating potential complications.

## Conclusions

This case highlights the challenges associated with managing elderly patients with multiple comorbidities, especially in the context of severe bradycardia and hypotension. The patient's complex medical history, combined with her age and the severity of her condition, made it difficult to effectively manage her care. Despite the best efforts of the medical team, the patient's condition continued to deteriorate, ultimately resulting in a fatal outcome.

This case underscores the importance of early identification and management of bradycardia and hypotension in elderly patients with multiple comorbidities. Additionally, this case highlights the need for comprehensive care plans for elderly patients with complex medical histories, including regular monitoring, timely interventions, and palliative care when appropriate.
